# PINK1 and Parkin Ameliorate the Loss of Motor Activity and Mitochondrial Dysfunction Induced by Peripheral Neuropathy-Associated HSPB8 Mutants in *Drosophila* Models

**DOI:** 10.3390/biomedicines11030832

**Published:** 2023-03-09

**Authors:** Kyong-hwa Kang, Ji Eun Han, Hyunjin Kim, Sohee Kim, Young Bin Hong, Jeanho Yun, Soo Hyun Nam, Byung-Ok Choi, Hyongjong Koh

**Affiliations:** 1 Department of Pharmacology, Dong-A University College of Medicine, Busan 49201, Republic of Korea; 2 Neuroscience Translational Research Solution Center, Dong-A University College of Medicine, Busan 49201, Republic of Korea; 3 Department of Translational Biomedical Sciences, Dong-A University College of Medicine, Busan 49201, Republic of Korea; 4 Peripheral Neuropathy Research Center, Dong-A University College of Medicine, Busan 49201, Republic of Korea; 5 Department of Biochemistry, Dong-A University College of Medicine, Busan 49201, Republic of Korea; 6 Department of Neurology, Sungkyunkwan University School of Medicine, Seoul 06351, Republic of Korea

**Keywords:** Charcot–Marie–Tooth disease, peripheral neuropathy, HSPB8, *Drosophila*, PINK1, parkin

## Abstract

Charcot–Marie–Tooth disease (CMT) is a group of inherited peripheral nerve disorders characterized by progressive muscle weakness and atrophy, sensory loss, foot deformities and steppage gait. Missense mutations in the gene encoding the small heat shock protein HSPB8 (HSP22) have been associated with hereditary neuropathies, including CMT. HSPB8 is a member of the small heat shock protein family sharing a highly conserved α-crystallin domain that is critical to its chaperone activity. In this study, we modeled *HSPB8* mutant-induced neuropathies in *Drosophila*. The overexpression of human HSPB8 mutants in *Drosophila* neurons produced no significant defect in fly development but led to a partial reduction in fly lifespan. Although these *HSPB8* mutant genes failed to induce sensory abnormalities, they reduced the motor activity of flies and the mitochondrial functions in fly neuronal tissue. The motor defects and mitochondrial dysfunction were successfully restored by *PINK1* and *parkin*, which are Parkinson’s disease-associated genes that have critical roles in maintaining mitochondrial function and integrity. Consistently, kinetin riboside, a small molecule amplifying PINK1 activity, also rescued the loss of motor activity in our *HSPB8* mutant model.

## 1. Introduction

Charcot–Marie–Tooth disease (CMT), also known as hereditary motor and sensory neuropathy, is the most common inherited peripheral neuron disorder [1]. CMT is a group of genetically and clinically heterogeneous disorders characterized by progressive muscle weakness and atrophy, foot deformities, steppage gait and sensory loss [2]. The disease is conventionally divided into a demyelinating form (referred to as CMT1) and an axonal defective form (CMT2). CMT1 shows markedly reduced nerve conduction velocities (NCVs), whereas CMT2 exhibits slightly reduced or normal NCVs [3,4]. In addition, sensory signs are occasionally lacking in CMT2 patients, so it is difficult to distinguish CMT2 from distal hereditary motor neuropathy (dHMN), which looks like axonal CMT but does not show sensory abnormalities [5].

To date, dozens of genes have been found to be linked with CMT2. These genes encode proteins with various functions, including axonal transport, protein folding, mitochondrial function, RNA metabolism and cation channel activity [6]. Among them, mutations in the small heat shock protein B8 (HSPB8) gene have been associated with CMT2, dHMN and distal myopathy diseases involving motor neurons and/or muscle cells [7,8,9,10,11,12]. HSPB8 (also called HSP22) is a small heat shock protein that has multiple cellular functions, including chaperone, oxidative stress response and anti-apoptosis activities [13]. The major disease-associated mutations of HSPB8 are missense mutations of the lysine 141 residue in the α-crystallin domain, which is critical to its chaperone activity [11,12,14]. Although several cell and mouse models expressing mutant HSPB8 have been developed and studied, it is not yet clear how a mutant HSPB8 induces neuronal and muscular phenotypes and whether a common pathogenesis underlies these diseases.

In this study, we introduced human *HSPB8* mutant genes into *Drosophila*. Although these *HSPB8* mutant genes produced no obvious defect in fly development, they induced a loss of fly motor activity and decreased mitochondrial activity and remodeling in fly neuronal tissues. Surprisingly, these *HSPB8* mutant-induced defects were restored by PINK1 and Parkin, which have critical roles in mitochondrial quality control [15,16]. Moreover, kinetin riboside, an activator of PINK1 [17], also rescued the loss of motor activity in our *HSPB8* mutant model, further confirming our findings.

## 2. Materials and Methods

### 2.1. Drosophila Strains

Human HSPB8 cDNA was obtained from Origene Technologies (Rockville, MD, USA). HSPB8 K141T and HSPB8 K141E mutant cDNAs were generated by a QuikChange™ site-directed mutagenesis kit (Agilent Technologies, Santa Clara, CA, USA) using the following primer pairs: HSPB8 K141T F (ctg cag gaa gct gga ttt tcg ttg tga agt tct tag aaa ca) and K141T R (tgt ttc taa gaa ctt cac aac gaa aat cca gct tcc tgc ag), HSPB8 K141E F (gaa gct gga ttt tct ctg tga agt tct tag aaa caa tgc cac c) and K141E R (ggt ggc att gtt tct aag aac ttc aca gag aaa atc cag ctt c). The wild-type and mutant HSPB8 cDNAs were cloned into the pACU2 vector and microinjected into yw;PBac y[+]-attP-3B VK00001 embryos. elav-GAL4 (BDSC_458), ppk-GAL4 (BDSC_32078), OK371-GAL4 (BDSC_26160), D42-GAL4 (BDSC_8816) and UAS-lacZ (BDSC_8530) lines were purchased from the Bloomington Stock Center (Bloomington, MN, USA). UAS-PINK1, UAS-Parkin and UAS-mt-Keima flies were generated as described previously [15,18].

### 2.2. Lifespan Assays

For the lifespan analysis, three groups of 30 flies (*n* = 90) were transferred to new food vials and checked for their survival every 3 or 4 days at 25 °C with a 12:12 h light–dark cycle. The Kaplan–Meier estimator and log-rank test were performed on the pooled cumulative survival data via the Online Application Survival Analysis 2 (OASIS2) web page (http://sbi.postech.ac.kr/oasis2; accessed on 21 March 2021).

### 2.3. Western Blots

To check HSPB8 expression, 20 male fly heads were homogenized in cell lysis buffer as previously described [19]. After purification, the lysates were boiled in SDS sample buffer at 95 °C. The samples were electrophoresed on 12% SDS–PAGE gels, and blotted to a nitrocellulose membrane. The membranes were blocked for 30 min and probed with an anti-HSPB8 antibody (1:1000, Cell Signaling Technology, Danvers, MA, USA) or anti-b-actin antibody (1:1000, Santa Cruz Biotechnology, Dallas, TX, USA) as previously described [19]. Membrane-bound antibodies were detected using the Odyssey XF imaging system (LI-COR Inc, Lincoln, NE, USA) at the Neuroscience Translational Research Solution Center (Busan, South Korea).

### 2.4. Larval Thermal Nociception Assays

Larval thermal nociception assays were performed as previously described [20]. In brief, the third instar larvae (120 h after egg laying) were washed with distilled water and placed on a petri dish. After 10 s of acclimatization, the larval abdominal A4-A5 segments were touched with a thermal probe whose temperature was controlled by a microprocessor control unit. The time required to provoke the aversive corkscrew-like rolling response was assessed as the withdrawal latency up to the 20-s cutoff. For each genotype, at least 50 larvae were evaluated, and the results are presented as the mean values (±SD).

### 2.5. Climbing Assays

The flies were divided into 15 size groups and incubated for 1 h in test vials at 25 °C for acclimatization. The number of climbing flies within 10 s was counted after tapping the flies down to the bottom. For each group, 10 trials were conducted, and the climbing score (percentage ratio of the number of climbing flies to the total number) was calculated. The average climbing score (±SD) was obtained for four or five independent tests.

### 2.6. Video-Tracked Behavioral Analysis

An adult fly was acclimated in a transparent petri dish (2 mm in height and 60 mm in diameter) at 25 °C for 30 min. Next, its free movement was recorded for 3 min using a digital video camera. The video file was converted to a MATLAB (The MathWorks Inc., Natick, MA, USA) file using Ctrax (Caltech, Pasadena, CA, USA), and the mean speed and trajectory path were calculated. The average speed (± SD) was obtained from five independent tests.

### 2.7. Measurement of Mitochondrial Membrane Potential

To measure the mitochondrial membrane potential, larval ventral nerve cords were dissected in PBS and incubated with 2.5 nM tetramethylrhodamine methyl ester (TMRM, Molecular Probes, Eugene, OR, USA) for 30 min in PBS containing 0.1% Triton X-100. A confocal analysis of TMRM fluorescence was performed with a Zeiss LSM 800 laser scanning confocal microscope (Carl Zeiss, Jena, Germany) located at the Neuroscience Translational Research Solution Center. The TMRM signal intensity was measured using Zeiss Zen software(version 3.4). The average TMRM fluorescence intensity (±SD) was calculated for six independent samples.

### 2.8. Measurement of Mitophagy Levels

The mitophagy levels were measured using the mitochondria-targeted fluorescent probe mt-Keima as previously described [18]. Larval ventral nerve cords expressing mt-Keima were dissected in PBS and examined with a Zeiss LSM 800 laser scanning confocal microscope located at the Neuroscience Translational Research Solution Center. mt-Keima fluorescence was imaged with two sequential excitation laser beams (488 nm and 555 nm) using a 595–700 nm emission bandwidth. The mt-Keima confocal images were analyzed using Zeiss Zen software on a pixel-by-pixel basis. The average mitophagy level (±SD) was calculated for 10 independent samples.

### 2.9. Statistical Analyses

To compare three or more groups, we used the Sidak test following a one-way ANOVA. For two-group comparisons, we used the two-tailed Student’s *t* test. A *p* value of <0.05 was considered statistically significant.

### 2.10. Genotypes

*Elav* (*elav-GAL4/+*); *elav HSPB8^WT^* (*elav-GAL4/+*; *UAS-HSPB8^WT^/+*); *elav HSPB^K141T^* (*elav-GAL4/+*; *UAS-HSPB8^K141T^/+*); *elav HSPB^K141E^* (*elav-GAL4/+*; *UAS-HSPB8 ^K141E^/+*); *ppk* (*ppk*-GAL4/+); *ppk HSPB8^WT^* (*ppk*-GAL4/*UAS-HSPB8^WT^*); *ppk HSPB^K141T^* (*ppk*-GAL4/*UAS-HSPB8^K141T^*); *ppk HSPB^K141E^* (*ppk*-GAL4/*UAS-HSPB8^K141E^*); *D42* (*D42-GAL4*/+); *D42 HSPB8^WT^* (*UAS-HSPB8^WT^/+*; *D42-GAL4/+*); *D42 HSPB^K141T^* (*UAS-HSPB^K141T^/+*; *D42-GAL4/+*); *D42 HSPB^K141E^* (*UAS-HSPB8^K141E^/+*; *D42-GAL4/+*); *elav HSPB^K141T^ PINK1* (*elav-GAL4/+*; *UAS-HSPB8^K141T^/UAS-PINK1*); *elav HSPB^K141T^ Parkin* (*elav-GAL4/+*; *UAS-HSPB8^K141T^/UAS-Parkin*); *elav HSPB^K141E^ PINK1* (*elav-GAL4/+*; *UAS-HSPB8^K141E^/UAS-PINK1*); *elav HSPB^K141E^ Parkin* (*elav-GAL4/+*; *UAS-HSPB8^K141E^/UAS-Parkin*); *elav HSPB8^WT^ mt-Keima* (*elav-GAL4/+*; *UAS-HSPB8^WT^/+*; *UAS-mt-Keima/+*); *elav HSPB^K141T^ mt-Keima* (*elav-GAL4/+*; *UAS-HSPB8^K141T^/+*; *UAS-mt-Keima/+*); *elav HSPB^K141E^ mt-Keima* (*elav-GAL4/+*; *UAS-HSPB8^K141E^/+*; *UAS-mt-Keima/+*); *elav HSPB^K141T^ PINK1 mt-Keima* (*elav-GAL4/+*; *UAS-HSPB8^K141T^/UAS-PINK1*; *UAS-mt-Keima/+*); *elav HSPB^K141T^ Parkin mt-Keima* (*elav-GAL4/+*; *UAS-HSPB8^K141T^/UAS-Parkin*; *UAS-mt-Keima/+*); *elav HSPB^K141E^ PINK1 mt-Keima* (*elav-GAL4/+*; *UAS-HSPB8^K141E^/UAS-PINK1*; *UAS-mt-Keima/+*); *elav HSPB^K141E^ Parkin mt-Keima* (*elav-GAL4/+*; *UAS-HSPB8^K141E^/UAS-Parkin*; *UAS-mt-Keima/+*); *OK371 HSPB8^WT^ mt-Keima* (*OK371-GAL4/UAS-HSPB8^WT^*; *UAS-mt-Keima/+*); *OK371 HSPB^K141T^ mt-Keima* (*OK371-GAL4/UAS-HSPB8^K141T^*; *UAS-mt-Keima/+*); *OK371 HSPB^K141E^ mt-Keima* (*OK371-GAL4/UAS-HSPB8^K141E^*; *UAS-mt-Keima/+*); *OK371 HSPB^K141T^ PINK1 mt-Keima* (*OK371-GAL4/UAS-HSPB8^K141T^*; *UAS-PINK1*/*UAS-mt-Keima*); *OK371 HSPB^K141T^ Parkin mt-Keima* (*OK371-GAL4/UAS-HSPB8^K141T^*; *UAS-Parkin*/*UAS-mt-Keima*); *OK371 HSPB^K141E^ PINK1 mt-Keima* (*OK371-GAL4/UAS-HSPB8^K141E^*; *UAS-PINK1*/*UAS-mt-Keima*); *OK371 HSPB^K141E^ Parkin mt-Keima* (*OK371-GAL4/UAS-HSPB8^K141E^*; *UAS-Parkin/UAS-mt-Keima*); *elav HSPB^WT^ lacZ* (*elav-GAL4/+*; *UAS-HSPB8^WT^/*+; *UAS-lacZ*/+); *elav HSPB^K141T^ lacZ* (*elav-GAL4/+*; *UAS-HSPB8^K141T^*/+; *UAS-lacZ*/+); *elav HSPB^K141E^ LacZ* (*elav-GAL4/+*; *UAS-HSPB8 ^K141E^*/+; *UAS-lacZ*/+); *elav HSPB^K141T^ Stv* (*elav-GAL4/+*; *UAS-HSPB8^K141T^/UAS-stv*); *elav HSPB^K141T^ stv^1/+^* (*elav-GAL4/+*; *UAS-HSPB8^K141T^/+*; *stv^1^/+*); *elav HSPB^K141E^ Stv* (*elav-GAL4/+*; *UAS-HSPB8 ^K141E^/UAS-stv*); *and elav HSPB^K141E^ stv^1/+^* (*elav-GAL4/+*; *UAS-HSPB8 ^K141E^/+*; *stv^1^/+*).

## 3. Results

### 3.1. Generation and Characterization of HSPB8 Transgenic Flies

**Figure 1:**
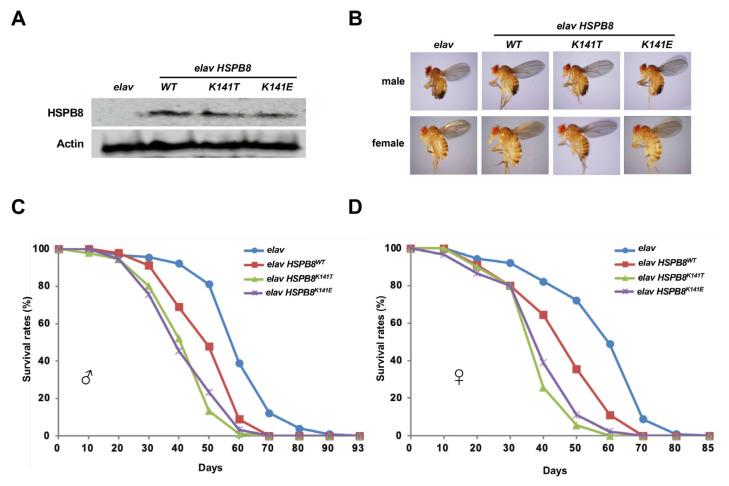
Characterization of HSPB8 transgenic flies. (**A**) Western blot analysis of HSPB8 proteins in *elav*-GAL4 (*elav*), HSPB8^WT^ (*elav HSPB8^WT^*), HSPB8^K141T^ (*elav HSPB8^K141T^*) and HSPB8^K141E^ (*elav HSPB8^K141E^*) expressing flies. Actin was used as a loading control. (**B**) Photographs of 5-day-old male and female flies. (**C**) Lifespan assays of male flies (log-rank test: *elav* vs. *elav HSPB8^WT^*: *p* < 0.001, *elav HSPB8^WT^* vs. *elav HSPB8^K141T^*, *elav HSPB8^WT^* vs. *elav HSPB8^K141E^*: *p* < 0.01, *n* = 90 per group). (**D**) Lifespan assays of female flies (log-rank test: *elav* vs. *elav HSPB8^WT^*: *p* < 0.001, *elav HSPB8^WT^* vs. *elav HSPB8^K141T^*, *elav HSPB8^WT^* vs. *elav HSPB ^K141E^*: *p* < 0.01, *n* = 90 per group). All lifespan assays were conducted at 25 °C and were repeated at least twice.

Missense mutations of the K141 residue of HSPB8 have been associated with dHMN and CMT2L disease, which mainly targets motor neurons [11,12,14]. We found three mutations of the K141 residue (K141N, K141E, and K141T) in *HSPB8* from a patient cohort with inherited peripheral neuropathy [21]. The vulnerability of motor neurons to mutated HSPB8 has been reported by overexpression studies in primary neuronal motor neuron cultures in which K141E and K141N HSPB8 caused neurite degeneration [22]. Although K141N mutant animal models have been recently developed [23], K141E and K141T mutant animal models are not yet available. To develop these two models, we generated fly lines with UAS transgenes of human wild type (*HSPB8^WT^*), K141T mutant (*HSPB8^K141T^*) and K141E mutant HSPB8 (*HSPB8^K141E^*). Using *elav*-*GAL4*, a pan-neuronal GAL4 driver, we expressed these HSPB8 transgenes specifically in *Drosophila* neurons. These transgenic flies showed no significant differences among them in their HSPB8 expression levels and successfully developed into adults (Figure 1A,B). In the lifespan assays, the expression of the wild-type and mutant HSPB8s caused a partial decrease in the lifespan, but no significant decrease in the survival rates was observed within 15 days (Figure 1C,D).

### 3.2. HSPB8 Transgenes Failed to Induce Sensory Phenotypes in the Drosophila Thermal Nociception Model

**Figure 2:**
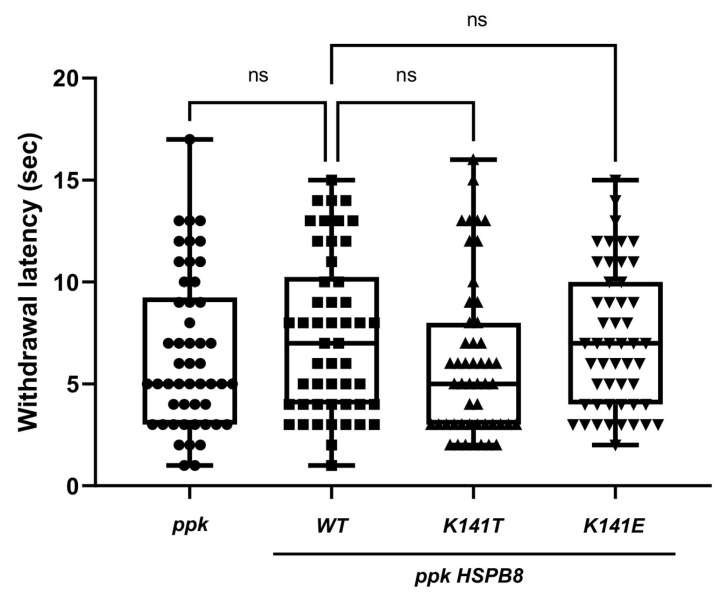
Thermal nociception of HSPB8 transgenic fly larvae. Average withdrawal latency of third instar larvae expressing HSPB8^WT^ (*ppk HSPB8^WT^*), HSPB8*^K141T^* (*ppk HSPB8^K141T^*) and HSPB8*^K141E^* (*ppk HSPB8^K141E^*) at 40 °C. Each data point demonstrates the withdrawal latency of an individual larva (*n* = 50 per group). Larvae with a *ppk-GAL4* driver (*ppk*) were used as controls. Significance was determined by Sidak test after one-way ANOVA (ns, not significant). Error bars indicate the mean ± SD.

Although most patients with K141 mutations show motor defects, some patients also report sensory symptoms. To test whether our mutant HSPB8 transgenes induced sensory defects, we adopted a recently developed thermal nociception assay using *Drosophila* larvae [20]. We expressed *HSPB8^WT^*, *HSPB8^K141T^* and *HSPB8^K141E^* transgenes in multidendritic sensory neurons using *ppk-GAL4*. Using a heat probe set to 40 °C, which was the temperature that induced the most dynamic thermal nociception response in our previous study [20], we touched the A4-A5 segment region of the larvae and measured the time required to induce the conventional rolling response to the noxious heat. In this assay, the *HSPB8 ^WT^* transgenic larvae demonstrated no significant difference in the mean withdrawal latency compared to the controls with only the *ppk*-GAL4 driver, *HSPB8^K141T^* and *HSPB8^K141E^* transgenes, showing that the HSPB8 transgenes did not induce sensory phenotypes in the *Drosophila* thermal nociception model (Figure 2).

### 3.3. Expression of Mutant HSPB8s in Drosophila Neurons Induced Loss of Motor Activity

**Figure 3:**
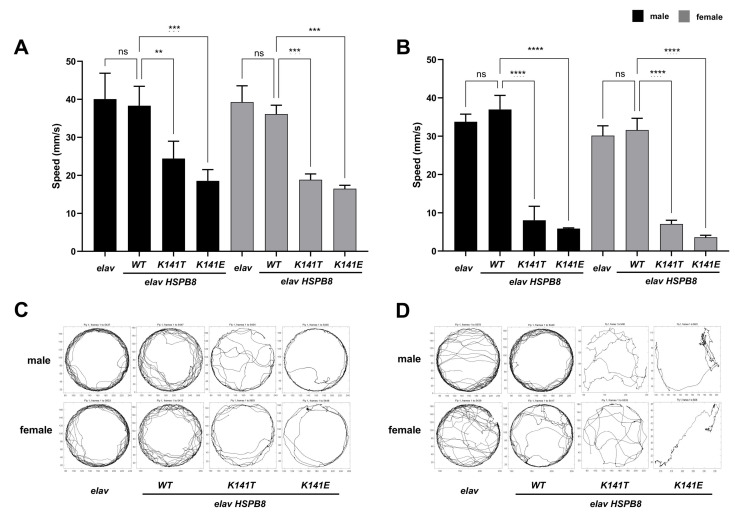
Expression of mutant HSPB8s in *Drosophila* neurons reduced motor performance. The following analyses were performed using *elav*, *elav HSPB8^WT^*, *elav HSPB8^K141T^* and *elav HSPB8^K141E^* male and female flies: (**A**,**B**) Comparison of the mean walking speeds for 5- (**A**) and 15-day-old flies (**B**) (*n* = 5 per group). (**C**,**D**) Movement trajectories of 5- (**C**) and 15-day-old flies (**D**). Significance was determined by Sidak test after one-way ANOVA (**, *p* < 0.01; ***, *p* < 0.001; ****, *p* < 0.0001; ns, not significant). Error bars indicate the mean ± SD.

To assess the effect of mutant HSPB8s on motor activity, we observed the behavior of the HSPB8 transgenic flies that were previously characterized (Figure 1). Their movement was recorded by a digital video camera, and the walking trajectories and speed were calculated by computer software. The 5-day-old male flies expressing HSPB8^WT^ demonstrated no meaningful change in motor activity compared to the controls expressing only the GAL4 protein (Figure 3A,B). In contrast, the HSPB8^K141T^- and HSPB8^K141E^-expressing flies displayed obvious defects in walking speed and trajectory (Figure 3A,B). The 5-day-old female flies also showed these mutant-specific motor defects (Figure 3A,B). When we measured the motor activities of 15-day-old flies, the *HSPB8^WT^* flies consistently showed no meaningful change in motor performance (Figure 3C,D). In contrast, the *HSPB8^K141T^* and *HSPB8^K141E^* flies showed a much more severe decline in their motor activities than the 5-day-old flies (Figure 3C,D).

Using the climbing assay, we double-checked the motor activity of our fly models. The climbing assay, which evaluates the climbing ability of the fly against gravity, is particularly useful in assessing locomotive deficits in fly models of movement disorders, such as Parkinson’s disease [15]. Although the expression of HSPB8^WT^ had no significant effect on climbing ability, the expression of HSPB8^K141T^ or HSPB8^K141E^ induced significantly decreased movement in 5-day-old male and female flies (Figure S1A). This loss of climbing ability was consistently observed in 15-day-old flies expressing HSPB8^K141T^ or HSPB8^K141E^ (Figure S1B).

Because motor neuron defects are the main symptoms in *HSPB8*-linked human pathologies, we checked the motor performance of transgenic flies expressing HSPB8 in motor neurons. Using the *D42-GAL4* driver, *HSPB8^WT^*, *HSPB8^K141T^* and *HSPB8^K141E^* transgenes were expressed in motor neurons. HSPB8^WT^ transgenic flies showed no significant difference in climbing ability compared to the control flies (Figure S1C,D). In contrast, HSPB8^K141T^ or HSPB8^K141E^ transgenic flies showed a strong decrease in motor activity compared to the control and *HSPB8^WT^* transgenic flies (Figure S1C,D). Moreover, flies expressing mutant HSPB8 transgenes under the *D42-GAL4* driver also showed an obvious decline in walking speed and trajectory in the video tracking analyses (Figure S2), confirming that the expression of human HSPB8 mutants in motor neurons consistently deteriorates motor performance in *Drosophila.*

### 3.4. PINK1 and Parkin Ameliorated Mitochondrial and Motor Defects in Mutant HSPB8 Transgenic Flies

**Figure 4:**
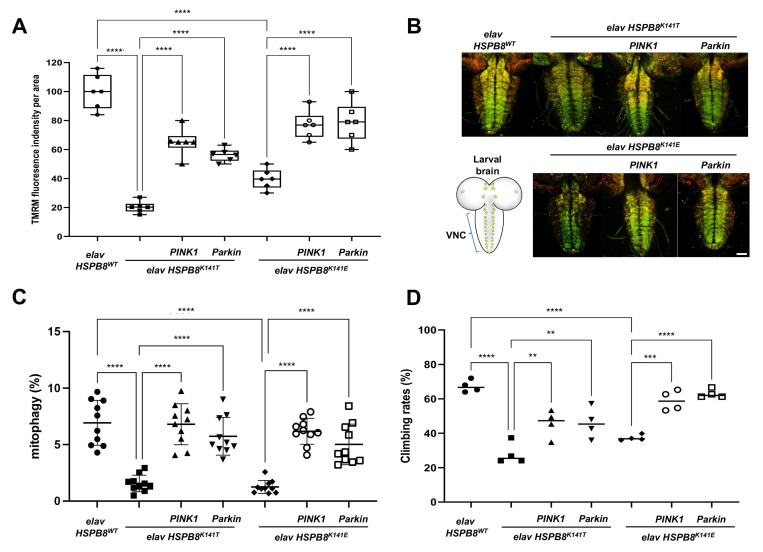
PINK1 and Parkin rescued mitochondrial defects induced by mutant HSPB8s. The following analyses were performed using wild-type HSPB8-expressing (*elav HSPB8^WT^*), mutant HSPB8-expressing (*elav HSPB8^K141T^*, *elav HSPB8^K141E^*), mutant HSPB8- and PINK1-expressing (*elav HSPB8^K141T^ PINK1*, *elav HSPB8^K141E^ PINK1*), mutant HSPB8- and Parkin-expressing (*elav HSPB8^K141T^ Parkin*, *elav HSPB8^K141E^ Parkin*) flies: (**A**) Mitochondrial membrane potential (ΔΨm) was measured in larval VNCs (*n* = 6 per group). (**B**) Representative mt-Keima fluorescence images of larval VNCs. (**C**) Quantitative analysis of mitophagy in larval VNCs (n = 10 per group). (**D**) Comparison of the climbing ability of 15-day-old male flies (*n* = 4 per group). Significance was determined by Sidak test after one-way ANOVA (**, *p* < 0.01; ***, *p* < 0.001; ****, *p* < 0.0001). Error bars indicate the mean ± SD. Scale bars: 50 μm.

Various studies have demonstrated the essential role of mitochondrial dysfunction in the pathophysiology of multiple neurodegenerative diseases [24]. Consistently, new roles are emerging for HSPB8 in maintaining mitochondrial function and integrity. After myocardial infarction, HSPB8 translocates to the mitochondrial inner membrane in rat hearts [25]. In a mouse heart failure model, increased HSPB8 expression stimulates mitochondrial oxidative phosphorylation, whereas its deletion has the opposite effect [26]. Moreover, mutant HSPB8 reduces the mitochondrial membrane potential in dermal fibroblasts from dHMN patients [7]. To test whether mutant HSPB8s could also affect the mitochondrial membrane potential in *Drosophila* neurons, we measured the membrane potential of the larval ventral nerve cord (VNC) (which is the functional equivalent of the vertebrate spinal cord that contains motor neurons) in HSPB8 transgenic flies with TMRM. The mitochondrial transmembrane potential was diminished in the VNCs of *HSPB8^K141T^* and *HSPB8^K141E^* mutant larvae compared with wild-type transgenics (Figure 4A), showing that our HSPB8 mutant flies successfully recapitulated patient phenotypes, such as mitochondrial dysfunction and the loss of motor activity. To further investigate the mitochondrial defects induced by mutant HSPB8, we examined the level of mitophagy, which is a critical mechanism for mitochondrial quality control, using the mitochondria-targeted fluorescent protein Keima (mt-Keima). At the physiological pH of the mitochondria (pH 8.0), mt-Keima shows an excitation peak at 440 nm. Within the acidic lysosome after mitophagy (pH 4.5), the excitation peak shifts to 586 nm [27]. Using this property, we measured the mitophagy activity in the VNC from HSPB8 transgenic larvae by coexpressing mt-Keima under the *elav-GAL4* driver. Surprisingly, the mitophagy levels were diminished in *HSPB8^K141T^* and *HSPB8^K141E^* mutant larvae compared with wild-type controls (Figure 4B,C). Moreover, when we expressed HSPB8 genes and mt-Keima in motor neurons using the *OK-371 GAL4* driver, *HSPB8^K141T^* and *HSPB8^K141E^* consistently suppressed mitophagy in the cell bodies of motor neurons located in the larval VNC (Figure S3A,B). Overall, these data suggested that the mutant HSPB8 proteins interrupt mitochondrial quality control and subsequently impair mitochondrial function.

To recover decreased mitochondrial activity and quality control in *HSPB8* mutant flies, we introduced *PINK1* and *parkin* transgenes. *PINK1* and *parkin* were originally cloned as familial Parkinson’s disease (PD) genes in human genetic analyses and have been identified as molecular guardians of mitochondria in fly genetic studies [15]. PINK1 is a mitochondrial kinase with a unique N-terminal mitochondrial targeting sequence [28]. Following the loss of mitochondrial membrane potential, PINK1 becomes stabilized on the depolarized mitochondria and recruits Parkin. Parkin, an E3 ubiquitin ligase, leads to the ubiquitylation of its substrates on the mitochondria and activates various mitochondrial remodeling processes, including mitophagy, to maintain mitochondrial function and integrity [16]. Consistent with their molecular roles, the expression of PINK1 and Parkin rescued the lost mitochondrial membrane potential (Figure 4A) and the decreased mitophagy levels (Figure 4B,C and Figure S3) in *HSPB8^K141T^* and *HSPB8^K141E^* transgenic flies. PINK1 and Parkin also rescued the decreased climbing ability (Figure 4D), walking speed (Figure S4A) and movement trajectory (Figure S4B) in both *HSPB8* mutant *Drosophila* models. In addition, the introduction of the *UAS*-*lacZ* transgene failed to rescue the locomotive defects in *HSPB8^K141T^* (Figure S5A) or *HSPB8^K141E^* mutant flies (Figure S5B), ruling out the possibility that the observed rescue may result from a weakened expression of mutant HSPB8s by increased UAS promoters from PINK1 or parkin transgenes. Overall, this rescue of motor defects accompanied by the restoration of mitochondrial activity and mitophagy suggested that mitochondrial dysfunction has a critical role in the pathogenesis of *HSPB8* mutation-induced neuropathies.

### 3.5. Kinetin Riboside Restored Locomotor Activity in Mutant HSPB8 Transgenic Flies

**Figure 5:**
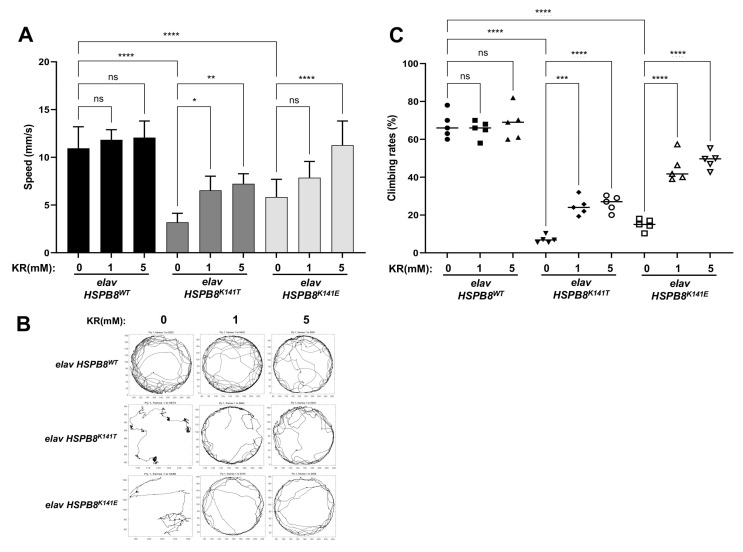
KR treatment ameliorated the loss of motor activity in mutant HSPB8 transgenic flies. (**A**,**B**) Comparison of the mean walking speeds (**A**) and movement trajectories (**B**) for 15-day-old *elav HSPB8^WT^*, *elav HSPB8^K141T^* and *elav HSPB8^K141E^* male flies treated with vehicle (0 mM) or KR (1 and 5 mM). (**C**) Comparison of the climbing ability of 15-day-old male flies after KR treatment (*n* = 5 per group). Significance was determined by Sidak test after one-way ANOVA (*, *p* < 0.05; **, *p* < 0.01; ***, *p* < 0.001; ****, *p* < 0.0001; ns, not significant). Error bars indicate the mean ± SD.

Because PINK1 is associated with human pathology, there has been growing interest in the discovery of small molecular compounds that amplify the kinase activity of PINK1. Recent studies have reported that N6-furfuryl adenine riboside, also known as kinetin riboside (KR), can activate PINK1 in cells independent of mitochondrial depolarization [17]. Therefore, we tested whether KR administration also rescues motor defects in *HSPB8* mutant transgenic flies. We raised 1-day-old flies on either fly food with KR (1 mM or 5 mM) or vehicle alone for 14 days and assessed their locomotor activity using video tracking analysis (Figure 5A,B) and climbing assays (Figure 5C). The wild-type transgenic flies showed no differences in locomotor activity under the KR treatment compared to the vehicle-treated controls (Figure 5A–C), indicating no significant side effects of KR on the fly models. In the *HSPB8* mutant transgenic flies, KR markedly restored locomotor activity in a dose-dependent manner (Figure 5A–C). These pharmacological data confirmed that PINK1 activation can rescue *HSPB8* mutation-induced phenotypes, suggesting that KR has the potential to suppress *HSPB8*-linked pathogenesis.

## 4. Discussion

Recent studies have generated transgenic mouse models leading to the expression of the HSPB8 mutant protein or *HSPB8* gene knockout [23]. While mice expressing mutant HSPB8 showed motor deficits associated with the degeneration of peripheral nerves and muscle atrophy, corroborating patient data, *HSPB8* knockout mice demonstrated motor performances equivalent to those of wild-type controls, proving the toxic gain of the function of mutant HSPB8 protein [23]. However, these mouse models develop motor deficits from at least 9 months of age onward. Therefore, we developed HSPB8 mutant models using *Drosophila*, which has an average lifespan of 2–3 months. By using elav-GAL4, the wild-type HSPB8 and two mutant HSPB8 proteins (HSPB8^K141T^ and HSPB8^K141E^) were expressed specifically in *Drosophila* neurons. These *HSPB8* transgenic flies showed no meaningful change in survival rates within 20 days after eclosion (Figure 1). The motor activity of the flies was assessed using video tracking analysis and a climbing assay, and the *HSPB8^K141T^* and *HSPB8^K141E^* flies started to show motor defects at 5 days after eclosion (Figure 3 and Figure S1). These motor deficits were fully developed by 15 days after eclosion, showing that our fly *HSPB8* models took much less time than the mouse model to recapitulate patient symptoms.

Cell-based studies have reported that HSPB8 binds to Beclin2-associated anathogen 3 (BAG3) and participates in maintaining cellular proteostasis [29]. HSPB8 dimers bind to BAG3, which also interacts with another chaperone, HSP70. With the E3 ligase CHIP and the autophagy adapter p62, this chaperone complex degrades proteins to be cleared through the autophagy process called chaperone-assisted selective autophagy (CASA) [30]. Consistently, autophagy defects were observed in the muscle of 12-month-old *HSPB8* mutant mice and blood cells from dHMN patients with the *HSPB8^K141E^* mutation [23,31]. The *Drosophila* BAG3 ortholog Starvin (Stv) interacts with the components of the CASA complex and degrades damaged proteins in *Drosophila* muscle [32,33]. Moreover, Stv has been shown to bind human HSPB8 protein in biochemical studies [34]. However, when we expressed Stv in *HSPB8^K141T^* and *HSPB8^K141E^* flies, there were no significant changes in their motor defects (Figure S6). In addition, a reduction in the *stv* gene dosage did not induce any meaningful change in motor activity in our *HSPB8* flies (Figure S6). These results indicated that these HSPB8 K141 mutant transgenes induce motor abnormalities through the different molecular mechanism which is not associated with the CASA complex.

To develop effective strategies to rescue motor defects in our *HSPB8* mutant models, we reviewed the in vivo roles of HSPB8 in various studies. Recent studies have shown the protective role of HSPB8 against mitochondrial dysfunction in disease models. In rodent heart disease models, HSPB8 was shown to translocate to the mitochondrial inner membrane where it stimulated mitochondrial oxidative phosphorylation [35]. Mutant HSPB8 induced the loss of mitochondrial membrane potential in fibroblasts from dHMN patients [7]. Consistently, our *HSPB8* mutant model flies showed significant mitochondrial depolarization in the larval VNC (Figure 4A). Because mitochondrial dysfunction has been closely associated with the pathogenesis of various neurodegenerative diseases, we examined the relationship between motor defects and the loss of mitochondrial function in our *HSPB8* flies. When we introduced PINK1 and Parkin, which translocate to depolarized mitochondria and remodel them to maintain mitochondrial function and integrity, these two genes successfully rescued mitochondrial depolarization and the motor deficits in *HSPB8^K141T^* and *HSPB8^K141E^* transgenic flies, suggesting that mitochondrial dysfunction has an essential role in *HSPB8* mutation-induced neuropathy (Figure 4A,D). Furthermore, we observed decreased mitophagy activity in motor neurons located in the VNC from the *HSPB8* mutant models and rescued the impaired mitophagy by introducing PINK1 and Parkin (Figure 4B,C and Figure S3). Consistent with our data, Li et al. reported that HSPB8 induces neuroprotective mitophagy against oxygen–glucose deprivation/reperfusion injury [36]. These data suggest that mutant HSPB8 interrupts mitophagy, the major mitochondrial quality control process, and subsequently induces mitochondrial dysfunction, which is critically linked to human pathology. Interestingly, the loss of mitophagy was observed in the soma, indicating that mitochondrial quality control failures in the cell body distribute damaged mitochondria to the axons and neuromuscular junction (NMJ)s of motor neurons and subsequently decrease motor neuron function in *HSPB8* mutant flies. Consistently, depolarized mitochondria were observed in the axons and NMJs in motor neurons from the *PINK1*-deficient flies [37], further supporting this idea. In addition, these results raise the possibility that HSPB8 is involved in PINK1-Parkin-mediated mitophagy. Therefore, we introduced *HSPB8* wild-type and mutant transgenes into *PINK1* and *Parkin* model flies but failed to observe any genetic interactions between them. Further studies are needed to clarify the molecular mechanism underlying the loss of mitophagy and mitochondrial activity induced by mutant HSPB8s.

The majority of clinically relevant PINK1 mutations abrogate its catalytic activity and prevent the induction of mitophagy upon mitochondrial damage, suggesting that the kinase activity of PINK1 is critical to the prevention of neurodegeneration [38]. This idea has been verified in *Drosophila PINK1* null mutants in which the kinase-inactive mutant of PINK1 failed to rescue neurodegeneration but the wild-type gene could [39]. This finding demonstrated the therapeutic potential of PINK1/Parkin pathway activation and initiated the development of PINK1-activating small molecules. Most reported PINK1-activating compounds act indirectly by causing a loss of mitochondrial membrane potential. These agents, including carbonyl cyanide m-chlorophenyl hydrazone (CCCP) and valinomycin, have facilitated the study of PINK1 signaling [16]; however, their cellular toxicity has limited their usage to activate PINK1 in vivo. In 2013, Hertz et al. discovered that PINK1 acts on neosubstrate N6-furfuryl ATP (kinetin triphosphate, KTP) with greater catalytic efficiency than its endogenous substrate, ATP [40]. The authors also reported that kinetin, the metabolic precursor of KTP, can be taken up by cells and changed into the nucleotide triphosphate form, which accelerates Parkin recruitment to depolarized mitochondria in human neuronal cells [40]. Recently, KR, another metabolic precursor of KTP, demonstrated significant activation of PINK1, while a treatment with kinetin did not induce noticeable PINK1 activation in the absence of CCCP, a mitochondrial depolarizing agent [17]. When we treated our *HSPB8* model flies with KR, the *HSPB8^WT^* flies showed no meaningful changes in motor performance, indicating that KR did not have a toxic effect on the fly models. However, in *HSPB8^K141T^* and *HSPB8^K141T^* flies, motor defects were successfully rescued in a dose-dependent manner (Figure 5). These data pharmacologically confirmed that PINK1 activation is an effective strategy to ameliorate the motor defects induced by HSPB8 mutants and indicate KR as a putative drug candidate for the treatment of *HSPB8*-associated human pathology.

In conclusion, we generated *Drosophila* models of *HSPB8*-associated neuropathies. The expression of human HSPB8 mutants in *Drosophila* neurons induced a loss of both motor activity and mitochondrial function. mt-Keima, a mitochondria-targeted fluorescent protein, revealed decreased mitophagy in neuronal tissues from *HSPB8* mutant transgenic flies. PINK1 and Parkin, which are critical regulators of mitochondrial quality control processes (including mitophagy), successfully rescued all of these defects. These data suggest that mitochondrial dysfunction has a critical role in the pathophysiology of neuropathies induced by HSPB8 mutations.

## Data Availability

Data are all contained within the article.

## Supplementary Materials

The following supporting information can be downloaded at: https://www.mdpi.com/article/10.3390/biomedicines11030832/s1, Figure S1: HSPB8 mutant genes decreased the climbing ability in *Drosophila*; Figure S2: Expression of mutant HSPB8 in motor neurons induced motor defects in *Drosophila*; Figure S3: PINK1 and Parkin rescued decreased mitophagy levels in motor neurons expressing mutant HSPB8s; Figure S4: PINK1 and Parkin rescued motor defects in *HSPB8* mutants; Figure S5: The expression of lacZ failed to rescue motor defects in *HSPB8* mutants; Figure S6: The expression and gene dosage reduction of *stv* failed to induce a meaningful change in motor activity in *HSPB8* mutants.
