# Supplementary material for: PINK1 and Parkin Ameliorate the Loss of Motor Activity and Mitochondrial Dysfunction Induced by Peripheral Neuropathy-Associated HSPB8 Mutants in Drosophila Models
Source: Biomedicines. 2023 Mar 9;11(3):832. doi: 10.3390/biomedicines11030832 (PMC10045361; doi:10.3390/biomedicines11030832)
Supplement: Supplementary file 1 [file biomedicines-11-00832-s001.zip › biomedicines-2248140-supplementary.pdf]

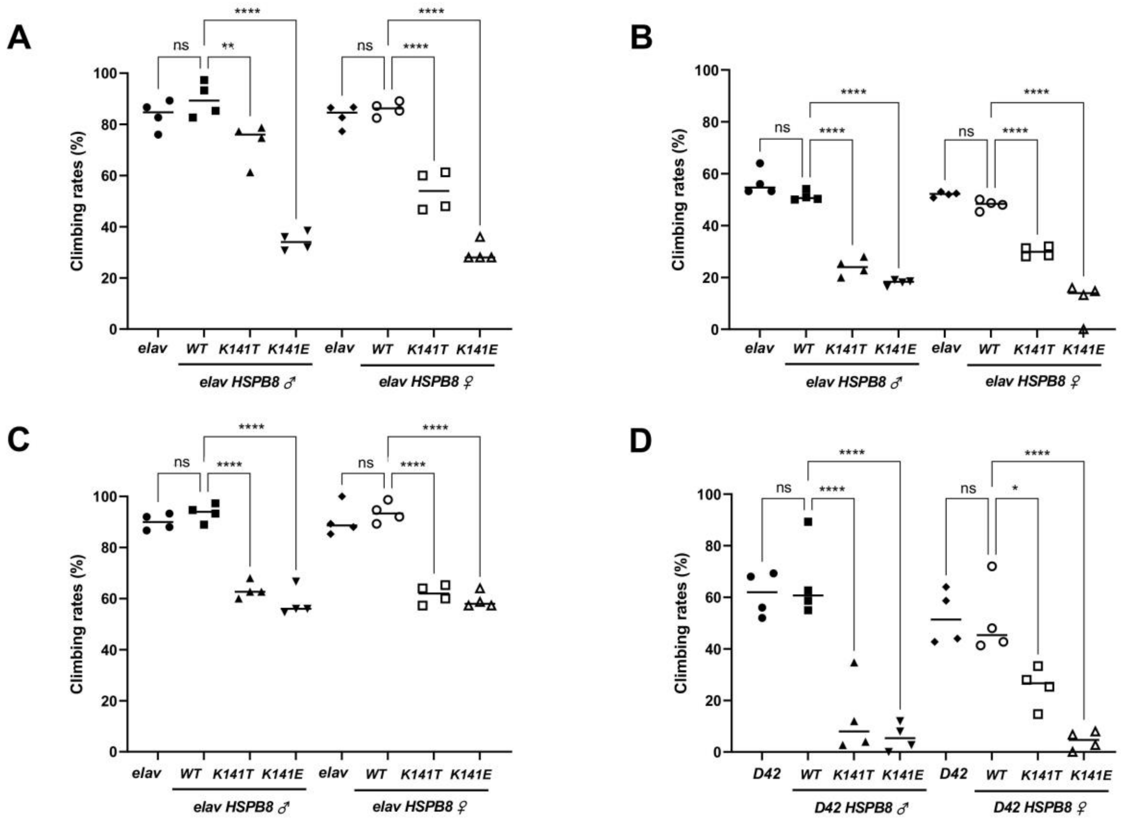

Figure S1. HSPB8 mutant genes decreased the climbing ability in *Drosophila*. (**A**) and (**B**) Comparison of climbing ability of 5- (**A**) and 15-day-old (**B**) *elav*, *elav HSPB8*^{WT}, *elav HSPB8*^{K141T} and *elav HSPB8*^{K141E} flies ($n = 4$ per group). (**C**) and (**D**) Comparison of climbing ability of 5- (**C**) and 15-day-old (**D**) flies expressing *D42*-GAL4 (*D42*), HSPB8^{WT} (*D42 HSPB8*^{WT}), HSPB8^{K141T} (*D42 HSPB8*^{K141T}) and HSPB8^{K141E} (*D42 HSPB8*^{K141E}) ($n = 4$ per group). Significance was determined by one-way ANOVA with Sidak correction (*, $p < 0.05$; **, $p < 0.01$; ****, $p < 0.0001$; NS, not significant). Error bars indicate the mean ± SD.

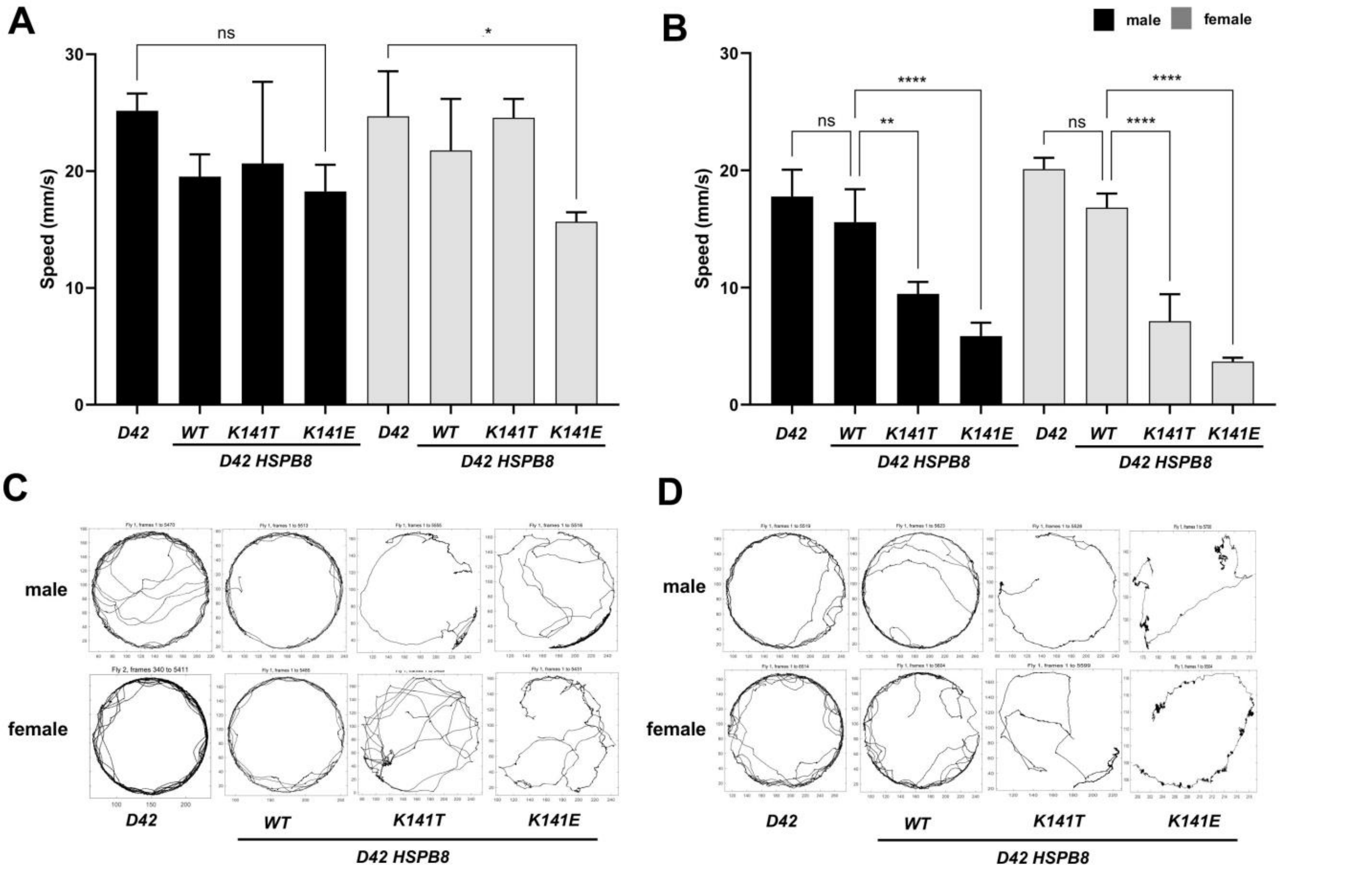

Figure S2. Expression of mutant HSPB8 in motor neurons induced motor defects in *Drosophila*. The following analyses were performed using 5- and 15-day-old flies expressing *D42*-GAL4 (*D42*), HSPB8^{WT} (*D42 HSPB8*^{WT}), HSPB8^{K141T} (*D42 HSPB8*^{K141T}) and HSPB8^{K141E} (*D42 HSPB8*^{K141E}): (**A**) and (**B**) Comparison of the mean walking speeds of (A) 5-day-old and (**B**) 15-day-old flies (n = 5 per group). (**C**) and (**D**) Movement trajectories of (**C**) 5- and (**D**) 15-day-old flies. Significance was determined by one-way ANOVA with Sidak correction (*, $p < 0.05$; **, $p < 0.01$; ****, $p < 0.0001$; NS, not significant). Error bars indicate the mean ± SD.

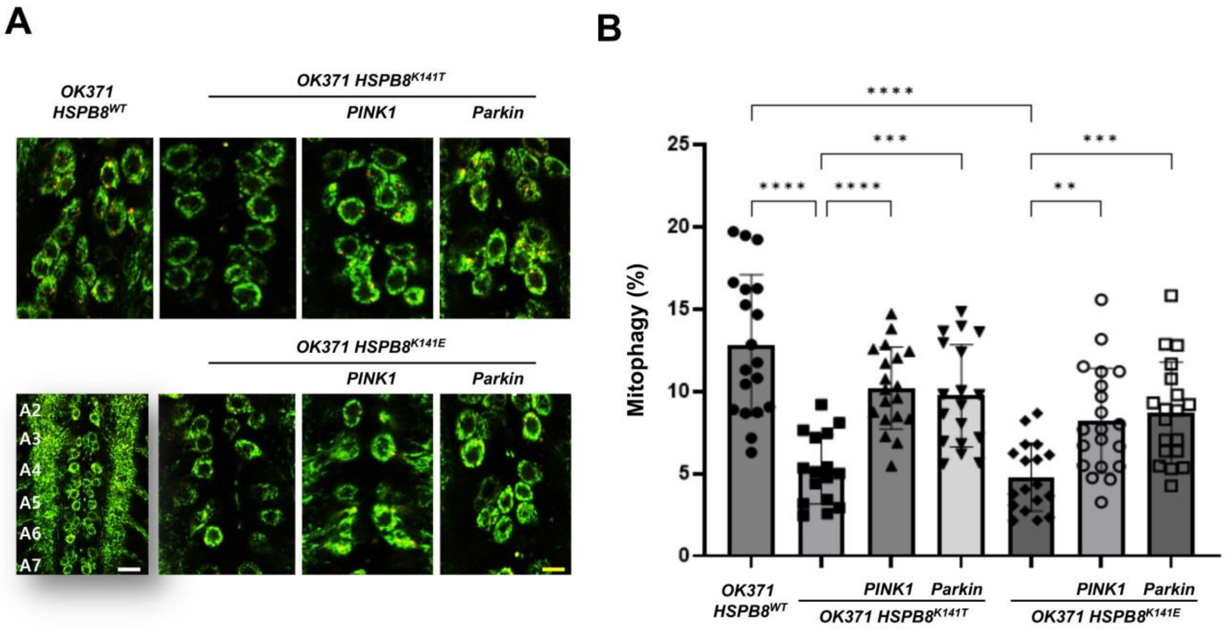

Figure S3. PINK1 and Parkin rescued decreased mitophagy levels in motor neurons expressing mutant HSPB8s. The following analyses were performed using wild-type HSPB8-expressing (*OK371 HSPB8*^{WT}), mutant HSPB8-expressing (*OK371 HSPB8*^{K141T}, *OK371 HSPB8*^{K141E}), mutant HSPB8- and PINK1-expressing (*OK371 HSPB8*^{K141T} *PINK1*, *OK371 HSPB8*^{K141E} *PINK1*) and mutant HSPB8- and Parkin-expressing (*OK371 HSPB8*^{K141T} *Parkin*, *OK371 HSPB8*^{K141E} *Parkin*) flies: (**A**) Representative mt-Keima fluorescence images of motor neurons located in A4-5 regions of larval VNC. Scale bars: 25 μm (white), 10 μm (yellow). (**B**) Quantitative analysis of mitophagy in motor neurons located in A4-5 regions of larval VNC (n = 15 per group). Significance was determined by one-way ANOVA with Sidak correction (**, $p < 0.01$; ***, $p < 0.001$; ****, $p < 0.0001$). Error bars indicate the mean ± SD.

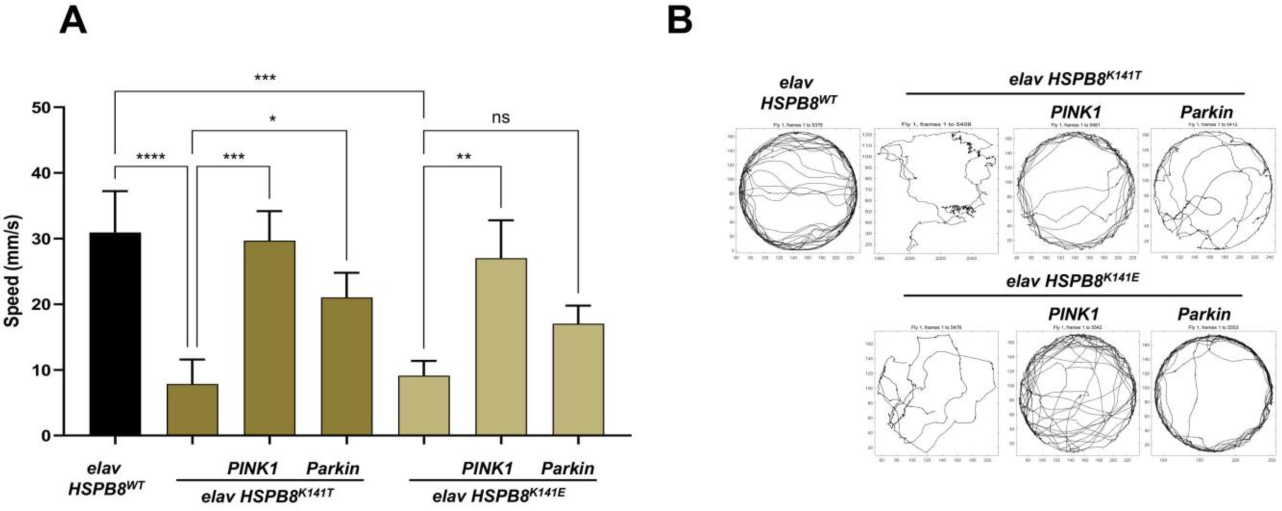

Figure S4. PINK1 and Parkin rescued motor defects in *HSPB8* mutants. The following analyses were performed using wild-type HSPB8-expressing (*elav* $HSPB8^{WT}$), mutant HSPB8-expressing (*elav* $HSPB8^{K141T}$, *elav* $HSPB8^{K141E}$), mutant HSPB8- and PINK1-expressing (*elav* $HSPB8^{K141T}$ *PINK1*, *elav* $HSPB8^{K141E}$ *PINK1*) and mutant HSPB8- and Parkin-expressing (*elav* $HSPB8^{K141T}$ *Parkin*, *elav* $HSPB8^{K141E}$ *Parkin*) flies: (**A**) Comparison of the mean walking speeds of 15-day-old male flies ($n = 5$ per group). (**B**) Movement trajectories of 15-day-old male flies. Significance was determined by one-way ANOVA with Sidak correction (*, $p < 0.05$; **, $p < 0.01$; ***, $p < 0.001$; ****, $p < 0.0001$; NS, not significant). Error bars indicate the mean ± SD.

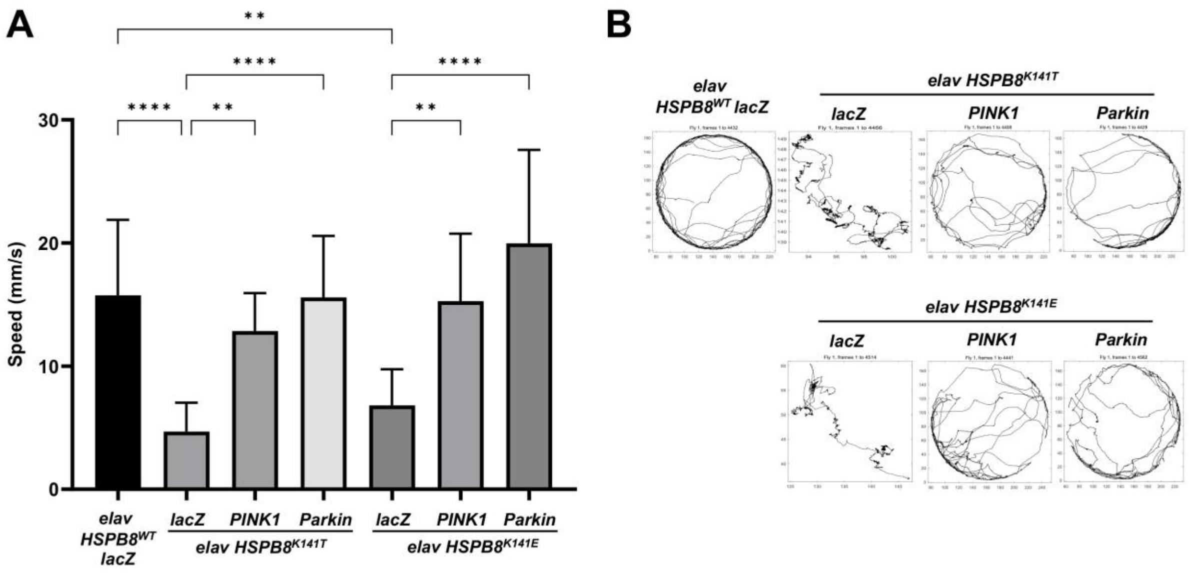

Figure S5. The expression of lacZ failed to rescue motor defects in *HSPB8* mutants. The following analyses were performed using wild-type HSPB8- and lacZ-expressing (*elav* $HSPB8^{WT}$ *lacZ*), mutant HSPB8- and lacZ-expressing (*elav* $HSPB8^{K141T}$, *elav* $HSPB8^{K141E}$), mutant HSPB8- and PINK1-expressing (*elav* $HSPB8^{K141T}$ *PINK1*, *elav* $HSPB8^{K141E}$ *PINK1*) and mutant HSPB8- and Parkin-expressing (*elav* $HSPB8^{K141T}$ *Parkin*, *elav* $HSPB8^{K141E}$ *Parkin*) flies: (**A**) Comparison of the mean walking speeds of 15-day-old male flies ($n = 5$ per group). (**B**) Movement trajectories of 15-day-old male flies. Significance was determined by one-way ANOVA with Sidak correction (**, $p < 0.01$; ****, $p < 0.0001$). Error bars indicate the mean ± SD.

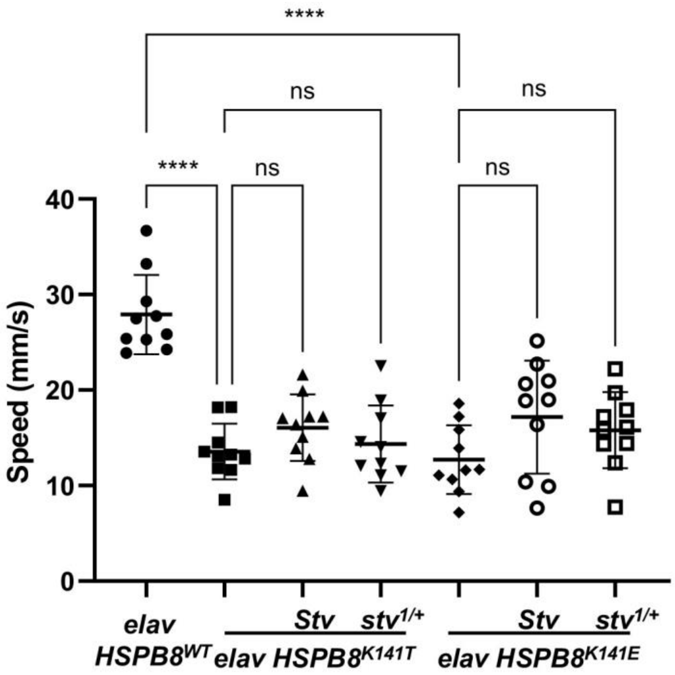

Figure S6. The expression and gene dosage reduction of *stv* failed to induce a meaningful change in motor activity in *HSPB8* mutants. Comparison of the mean walking speeds of 15-day-old male flies was performed using wild-type HSPB8-expressing (*elav HSPB8^{WT}*), mutant HSPB8-expressing (*elav HSPB8^{K141T}*, *elav HSPB8^{K141E}*), mutant HSPB8- and Stv-expressing (*elav HSPB8^{K141T} Stv*, *elav HSPB8^{K141E} Stv*) and mutant HSPB8-expressing flies with a heterozygous *stv* mutation (*elav HSPB8^{K141T} stv$^{1/+}$*, *elav HSPB8^{K141E} stv$^{1/+}$*) (n = 10 per group). Significance was determined by one-way ANOVA with Sidak correction (****, $p < 0.0001$; NS, not significant). Error bars indicate the mean ± SD.
